# Isolation of Allelochemicals from *Rhododendron capitatum* and Their Allelopathy on Three Perennial Herbaceous Plants

**DOI:** 10.3390/plants13182585

**Published:** 2024-09-15

**Authors:** Hang Yang, Yishan Zhao, Shaochong Wei, Xiaojun Yu

**Affiliations:** 1 College of Grassland Science, Gansu Agricultural University, Lanzhou 730070, China; yhang_gsau@163.com (H.Y.); xx99306015@163.com (Y.Z.); weisc@st.gsau.edu.cn (S.W.); 2 Key Laboratory of Forage Gerplasm Innovation and New Variety Breeding of Ministry of Agriculture and Rural Affairs (Co-Sponsored by Ministry and Province), Lanzhou 730070, China

**Keywords:** *Rhododendron capitatum*, shrub encroachment, allelopathy, allelochemical, alpine meadow

## Abstract

*Rhododendron capitatum* community expansion is a major threat to alpine meadow. Allelopathy is an important mediator in managing relationships between plants in natural ecosystems. However, allelopathy and specific allelochemicals of *R. capitatum* have not been studied yet. In this study, the allelopathy of the foliage litter of *R. capitatum* was explored on *Elymus nutans*, *Poa pratensis* and *Medicago ruthenica*, and the chemical composition and their allelopathy were studied. The results showed that the aqueous extract of the foliage litter of *R. capitatum* had an allelopathy of “low concentration promotion and high concentration inhibition” on the germination of *E. nutans*, *P. pratensis*, and *M. ruthenica*. Organic acids, fatty acids, terpenes, phenols, and phenolic acid compounds were identified, with Zanamivir (77.81%), alpha-linolenic acid (18%), Kaurenoic acid (23.50%), 4-hydroxyphenylglycolic acid (21.54%), and Quinic acid (28.24%) having the highest relative content, and all five compounds showed significantly inhibitory effects on seed germination and seedling growth of *E. nutans*, *P. pratensis*, and *M. ruthenica*, which further suggests that the five compounds are the critical allelochemicals for negative allelopathy of *R. capitatum*. These results highlight the crucial role of inhibitory allelopathy produced by *R. capitatum* in the establishment and expansion of its populations.

## 1. Introduction

Grasslands are an important component of terrestrial ecosystems, accounting for approximately 40% of the land area [1], and play a crucial role in maintaining ecological balance and providing valuable services to humans [2,3]. Global grasslands are currently experiencing widespread shrub encroachment due to warming ambient temperatures, rise in atmospheric carbon dioxide (CO_2_) concentrations, and overgrazing [4]. This rapid expansion is considered a major threat to grassland ecosystems [5], described as the dominance and coverage of shrub plants rapidly increasing leading to changes in grassland ecosystem status, such as abrupt changes in species composition, plant diversity, and soil moisture and nutrients [2,6,7]. As a result, studies of mechanisms of shrub encroachment in grassland ecosystems are receiving growing attention.

The growth of neighboring plants is affected by several different mechanisms, including allelopathy, competition, or a combination of both. Allelopathy refers to the inhibitory (or stimulatory) effect of plants producing and releasing allelochemicals that interfere with the establishment and growth of other organisms [8]. Plants release allelochemicals via decomposition, volatilization, leaching, and root exudation in soil or air [9]. Allelochemicals can directly or indirectly stimulate surrounding plants, such as directly interfering with the abilities of photosynthesis, respiration, enzyme synthesis and metabolism or indirectly affecting target plants by altering soil properties and microbial communities [10,11,12]. Living and dead plants can release allelochemicals to affect neighboring plants [13,14], and allelopathy can drive plant invasion and exacerbate grassland degradation [12]. Therefore, it is very meaningful to study shrub invasion and expansion from the perspective of allelopathy in grassland ecosystems.

Different types of allelochemicals are mainly classified into simple organic acids, terpenoids, unsaturated fatty acids, simple phenols, amino acids, tannins, aldehydes or ketones, lactones, straight-chain alcohols, naphthoquinones, quinone complex, polypeptides, alkaloids, glucosinolates, purines, and nucleotides based on structures and properties [15,16]. Various compounds have been found in plants and have inhibited seed germination and growth in other plants [17]. Palmitic acid, Ethyl palmitate, Linoleic acid, Ethyl linolenate, and Ethyl linoleate compounds from *Ipomoea batatas* had significant inhibitory effects on seeds germination and seedlings growth of *Lolium multiflorum* and *Phalaris minor* [18]. Syringic acid, tricin, acetin, syringoside, and diosmetin from *Avena fatua* inhibited the growth of seedlings and roots of *Triticum aestivum* at a concentration of 100 mg·kg^−1^ [19]. There are differences in the allelopathy potential due to the types and concentrations of allelochemicals released by plants, as well as the variety of allelochemicals among different plants. Studies on the isolation of compounds and the allelopathy potential in most plants still need to be supplemented.

*Rhododendron capitatum* belongs to the *Rhododendron* genus of the Rhododendron family [20,21] and is the main dominant species in the alpine meadows of the Qinghai–Tibet Plateau [22,23]. The alpine meadows are crucial for local herders’ livestock breeding and economic income, and the increase in shrub population is the main threat to the alpine meadow, where *Rhododendron* spp. is the main invasive shrub and is not favored by livestock [22,24]. In addition, *R. capitatum* is rich in a variety of compounds and used as a traditional Tibetan medicine [25]. However, studies on the allelopathic effects and the isolation of allelochemicals in *R. capitatum* are still scarce.

In this study, we aimed to investigate the potential allelopathy of *R. capitatum* on three dominant forage species in alpine meadows. We tested the effects of aqueous extracts of foliage litter of *R. capitatum* on seed germination and shoot and root growth of *Elymus nutans*, *Poa pratensis*, and *Medicago ruthenica*. Then, the relative contents of organic acids, fatty acids, terpenes, phenols, and phenolic acid compounds in the foliage of *R. capitatum* were tested, and the compounds with the highest relative content among the five types of compounds were selected to determine its effects on seed germination and seedling growth of *E. nutans*, *P. pratensis*, and *M. ruthenica*. The findings of this study will contribute to the understanding of the chemical drivers of the formation and expansion of *R. capitatum* shrubs in alpine meadows.

## 2. Results

### 2.1. Effects of Extracts of Rhododendron capitatum on Seed Germination and Seedling Growth of Elymus nutans, Poa pratensis, and Medicago ruthenica

**Figure 1:**
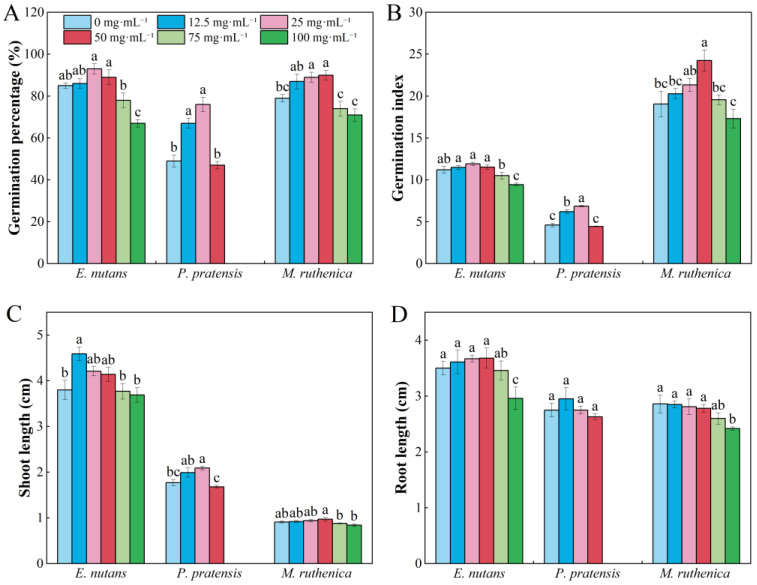
Effects of different concentrations of water extracts from *Rhododendron capitatum* foliage litter on seed germination (**A**,**B**) and seedling growth (**C**,**D**) of three species grown in alpine meadow in Qinghai–Tibet Plateau. Different lowercase letters above the bars indicate significant differences (*p* < 0.05) among concentrations under the same species. Values represent means ± SE.

The effect of aqueous extracts of *R. capitatum* foliage litter on the germination percents (GP) of *E. nutans*, *P. pratensis*, and *M. ruthenica* seeds showed a concentration effect. GP showed a unimodal increasing trend with the increase in extract concentration. *P. pratensis* seeds had no germination performance at concentrations of 75 mg·mL^−1^ and 100 mg·mL^−1^ (Figure 1A). The aqueous extracts of *R. capitatum* foliage litter significantly reduced the germination index (GI) of *E. nutans* at 100 mg·mL^−1^, while the GI of *M. ruthenica* showed a single peak change with increasing concentration, with a peak at 50 mg·mL^−1^ (Figure 1B). *R. capitatum* promoted the seedling length (SL) of *E. nutans* seedlings at a concentration of 25 mg·mL^−1^, while the SL of *P. pratensis* was significantly promoted at a concentration of 50 mg·mL^−1^ (Figure 1C). The root length (RL) of *E. nutans* and *M. ruthenica* was significantly inhibited at a concentration of 100 mg·mL^−1^ (Figure 1D). The extract of *R. capitatum* foliage litter showed a positive allelopathic effect on the GP, GI, SL, and RL of *E. nutans* at a concentration of 12.5–50 mg·mL^−1^, while an inhibitory effect was observed at a concentration of 50–100 mg·mL^−1^, and the allelopathy of *R. capitatum* on the RL of *M. ruthenica* had negative effects (Table S1).

### 2.2. Isolation of Allelochemicals from Foliage Litter of Rhododendron capitatum

There were eight types of organic acid compounds with a relative content of more than 1% in the foliage of *R. capitatum*. The relative contents of Zanamivir, Citric acid, and Allicin were 77.81%, 9.57%, and 3.39%, respectively (Table 1). The fatty acids and derivatives with relatively high content were alpha linolenic acid, 2-Hydroxyhexanoic acid, and cis-aconitic acid, and the relative contents were 18.00%, 9.05%, and 6.61%, respectively (Table 2). There were 19 terpenoid compounds with a relative content of more than 1%. The relative contents of Kaurenoic acid, 2-Angeloyl-9-(3-methyl-2E-pentenoyl)-2b,9a-dihydroxy-4Z,10(14)-octopadien-3-one, and Arjunolic acid were 23.50%, 10.98%, and 8.44%, respectively (Table 3). The relative contents of phenolic compounds 4-Hydroxyphenylglycolic acid, Epinephrine, and 6-Hydroxyshogaol were 21.54%, 17.67% and 15.49%, respectively (Table 4). The relative contents of phenolic compounds Quinic acid, Methylvanillate, and Propyl paraben were 28.24%, 18.13%, and 12.20%, respectively (Table 5).

### 2.3. Effects of Allelochemicals of Rhododendron capitatum on Seed Germination and Seedling Growth of Elymus nutans, Poa pratensis, and Medicago ruthenica

**Figure 2:**
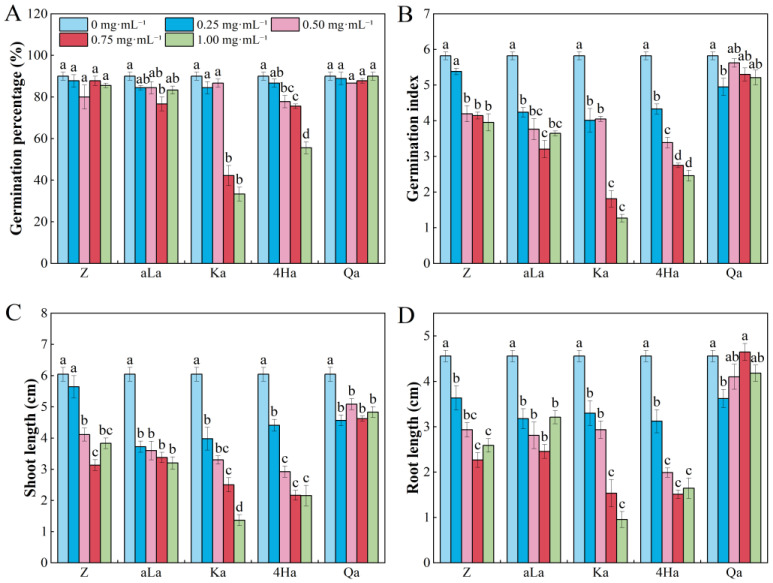
Effects of different concentrations of five compounds from *Rhododendron capitatum* foliage litter on seed germination (**A**,**B**) and seedling growth (**C**,**D**) of *Elymus nutans*. Different lowercase letters above the bars indicate significant differences (*p* < 0.05) among concentrations under the same compound. Z represents Zanamivir; aLa represents alpha-linolenic acid; Ka represents Kaurenoic acid; 4Ha represents 4-Hydroxyphenylglycolic acid; Qa represents Quinic acid. Values represent means ± SE.

Kaurenoic acid and 4-hydroxyphenylglycolic acid inhibited the germination of *E. nutans* seeds, showing a decreasing trend with increasing concentration of allelochemicals (Figure 2A). Zanamivir, alpha-linolenic acid, Kaurenoic acid, 4-hydroxyphenylglycolic acid, and Quinic acid all decreased the GI and SL of *E. nutans* seeds at concentrations of 0.25–1.0 mg·mL^−1^ (Figure 2B,C). Zanamivir, alpha-linolenic acid, Kaurenoic acid, and 4-hydroxyphenylglycolic acid reduced the RL of *E. nutans* seedlings (Figure 2D). Quinic acid at concentrations of 0.05 and 0.75 mg·mL^−1^ had a promoting allelopathic effect on the RL of *E. nutans* (Table S2).

**Figure 3:**
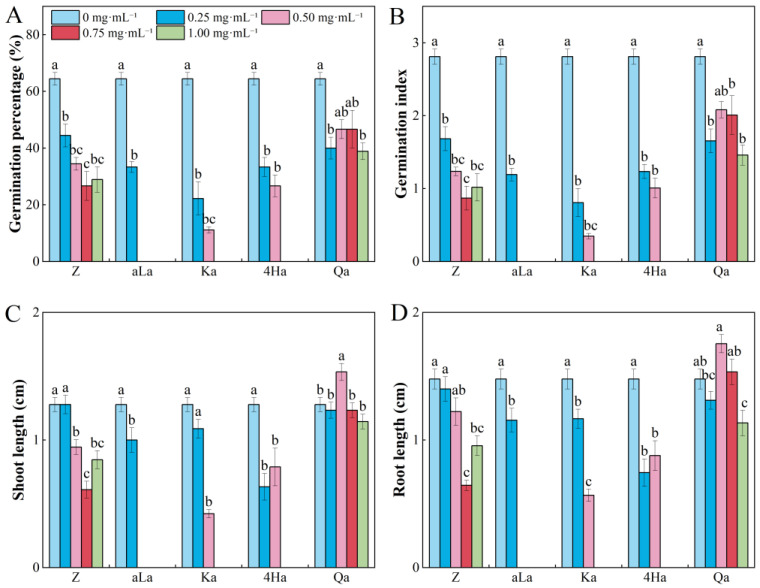
Effects of different concentrations of five compounds from *Rhododendron capitatum* foliage litter on seed germination (**A**,**B**) and seedling growth (**C**,**D**) of *Poa pratensis*. Different lowercase letters above the bars indicate significant differences (*p* < 0.05) among concentrations under the same compound. Z represents Zanamivir; aLa represents alpha-linolenic acid; Ka represents Kaurenoic acid; 4Ha represents 4-Hydroxyphenylglycolic acid; Qa represents Quinic acid. Values represent means ± SE.

Zanamivir, alpha-linolenic acid, Kaurenoic acid, 4-hydroxyphenylglycolic acid, and Quinic acid inhibited the GP and GI of *P. pratensis* seeds. Alpha-linolenic acid showed no germination trend under concentration of 0.5–1.0 mg·mL^−1^ (Figure 3A,B, Table S2). Zanamivir, alpha-linolenic acid, Kaurenoic acid, and 4-hydroxyphenylglycolic acid had inhibitory allelopathic effects on the SL and RL of *P. pratensis* seedlings, while Quinic acid promoted the SL and RL of *P. pratensis* seedlings at a concentration of 0.5 mg·mL^−1^ (Figure 3C,D, Table S2).

**Figure 4:**
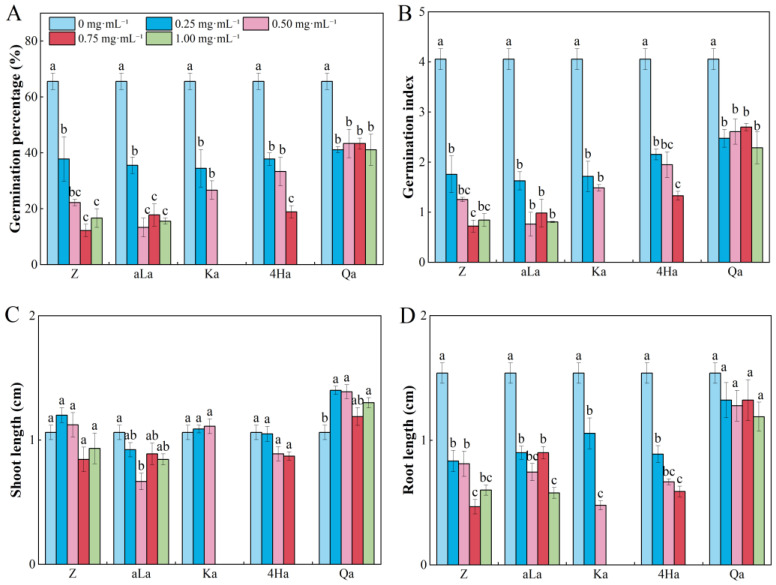
Effects of different concentrations of five compounds from *Rhododendron capitatum* foliage litter on seed germination (**A**,**B**) and seedling growth (**C**,**D**) of *Medicago ruthenica*. Different lowercase letters above the bars indicate significant differences (*p* < 0.05) among concentrations under the same compound. Z represents Zanamivir; aLa represents alpha-linolenic acid; Ka represents Kaurenoic acid; 4Ha represents 4-Hydroxyphenylglycolic acid; Qa represents Quinic acid. Values represent means ± SE.

Zanamivir, alpha-linolenic acid, Kaurenoic acid, 4-hydroxyphenylglycolic acid, and Quinic acid inhibited the GP, GI, and RL of *M. ruthenica*. Kaurenoic acid and 4-Hydroxyphenylglycolic acid at concentrations of 0.75–1.0 mg·mL^−1^ showed no germination behavior in *M. ruthenica* seeds (Figure 4A,B,D, Table S2). Low concentrations (0.25–0.5 mg·mL^−1^) of Zanamivir and Kaurenoic acid promoted the SL of *M. ruthenica*, while Quinic acid had a positive allelopathic effect on the SL of *M. ruthenica* seedlings at concentrations of 0.25–1.0 mg·mL^−1^ (Figure 4C).

## 3. Discussion

The *R. capitatum* is an evergreen shrub living in natural sites and mainly growing in alpine meadows, alpine grasslands, and wet grasslands at an altitude of 2500–4300 m [25]. Shrub invasion can result in plant community changes due to a reduction in perennial herbaceous plants in alpine meadows [5], as well as a redistribution of soil nutrient resources, such as the formation of “soil fertile islands” [26], and land pattern changes in mosaics with shrub patches have significantly affected landscape performance [27,28]. Allelopathy is known to affect individual plant performance, community structure, and plant invasion [29]. In this study, the aqueous extracts of *R. capitatum* foliage litter showed allelopathy on the germination and seedling growth of *E. nutans*, *P. pratensis*, and *M. ruthenica*, and the germination and seedling growth were promoted at low concentrations but also were inhibited to varying degrees at high concentrations of aqueous extracts. Previous studies have shown that the allelochemicals produced by donor plants have an allelopathy of “low concentration promotion and high concentration inhibition” on target plants, which is consistent with the results of this study [30,31]. This also indicates that the stimulating and inhibitory effects of the extract are a function of concentration [17], which may be because the lower concentration of the aqueous extract causes a slight disturbance to the organism, and self-repair and maintenance mechanisms are triggered, such as increased expression of cellular repair and protective proteins [32]. In addition, the results of this study indicate that 25 mg·mL^−1^ is the threshold concentration for the aqueous extract of *R. capitatum* to inhibit the growth of *E. nutans* and *P. pratensis*, while 50 mg·mL^−1^ is the threshold concentration for inhibiting the growth of *M. ruthenica*, which suggests that the allelopathic effects of the same donor plant will be differently expressed due to the different receptor plants [33].

In this study, the promotion and inhibition effects of *R. capitatum* on *E. nutans*, *P. pratensis*, and *M. ruthenica* confirm a significant dose–response relationship between *R. capitatum* shrubs and perennial herbaceous plants. Seed germination and seedling growth, as important processes for establishing plant communities and expanding, may be disrupted by various biotic and abiotic factors [34]. Therefore, this study’s results indicate that the release of allelochemicals from *R. capitatum* into the soil can affect the seed germination of *E. nutans*, *P. pratensis*, and *M. ruthenica* due to leaching. The germination of herbaceous plant seeds is inhibited when the concentration of allelochemicals reaches a threshold, which may affect the establishment of perennial plant communities such as *E. nutans*, *P. pratensis*, and *M. ruthenica*. This process may be beneficial for the further expansion and invasion of *R. capitatum* in alpine meadows [32].

*Rhododendron* contains a variety of chemical components with different structures. Previous studies have extracted terpenes, flavonoids, and volatile compounds from *Rhododendron* plants [21,23,25]. In this study, organic acids, fatty acids, terpenes, phenols, and phenolic acid compounds were observed in the foliage litter of *R. capitatum*, among which terpenes and phenolic acid compounds with a relative content greater than 1% were more abundant. The allelopathy of Zanamivir, alpha-linolenic acid, Kaurenoic acid, 4-hydroxyphenylglycolic acid, and Quinic acid was analyzed using standard compound addition methods. The five compounds showed varying degrees of inhibitory effects on the seed germination and seedling growth of *E. nutans*, *P. pratensis*, and *M. ruthenica*. This further confirms that *R. capitatum* contains allelochemicals that have a negative allelopathy on perennial herbaceous plants in alpine meadows.

Alpha-linolenic acid and Quinic acid have been reported as allelochemicals by previous reports [35,36], but many of the compounds in the present study have not been reported, especially in the *R. capitatum*. In this study, Kaurenoic acid and 4-hydroxyphenylglycolic acid showed a concentration-dependent allelopathy on *E. nutans*, significantly inhibiting seeds germination and seedlings growth with increasing concentration. The alpha-linolenic acid, Kaurenoic acid, and 4-hydroxyphenylglycolic acid completely inhibited the germination of *P. pratensis* seeds at the concentration thresholds, while *M. ruthenica* showed no signs of germination under higher concentrations of Kaurenoic acid and 4-hydroxyphenylglycolic acid; this suggests the heterogeneity of allelopathy of the same compounds on different target plants [37,38].

The seed bank is an important component for maintaining plant communities and vegetation reproduction in alpine meadows [39]. In this study, the allelochemicals in the foliage litter of *R. capitatum* significantly inhibited the germination of three target plant seeds. In addition, the alpha-linolenic acid, Kaurenoic acid, and 4-hydroxyphenylglycolic acid both significantly inhibited the shoot growth of *E. nutans* and *P. pratensis* seedlings, while Quinic acid inhibited the root growth of *M. ruthenica* but promoted shoot growth. This may be due to allelopathy affecting biological processes, such as cell division and elongation, oxidation and antioxidant systems, and protein and enzyme synthesis in seedlings [40]. On the one hand, this indicates that the allelopathy produced by *R. capitatum* reduces the productivity of perennial excellent forage in grasslands, while inhibiting the seed reproduction and seedling growth ability of herbaceous plants, which may be beneficial for the establishment of the *R. capitatum* community. On the other hand, the results indicate that the allelopathy of the same allelochemicals on different organs of the target plant are not completely consistent [41].

Grazing is the main utilization of alpine meadows, and *R. capitatum* is not preferred by livestock due to poor palatability [24]. Allelochemicals are present in the soil due to litter accumulation and leaching on grasslands, which may lead to an increase in the concentration of allelochemicals in the surrounding environment of the *R. capitatum* shrubs and inhibit the germination and growth of perennial herbaceous plants in the area. At the same time, rapid shrub expansion caused by climate warming may exacerbate the accumulation of allelochemicals in the environment, and the reported allelochemicals in this study have significance for studying shrub expansion driven by allelopathy. However, it is important to recognize whether the concentration threshold of allelochemicals has been reached to promote or inhibit the target plant. In our study, the concentration was set to elucidate the allelopathy under controlled conditions, Wen et al. [42] showed that the experimental concentration cannot fully reflect the true concentration of allelochemicals in the natural environment and that soil microbial degradation and synergistic processes may affect the differences in allelochemicals concentrations, whereas the current experiments on allelopathy under natural conditions are relatively rare [29]. At the same time, the synergistic effect produced by multiple allelochemicals may also be a major factor influencing allelopathy [43]. Therefore, natural conditions and multiple allelochemicals’ synergies should be considered to understand the ecological significance of allelopathy in future studies.

## 4. Materials and Methods

### 4.1. Collection of Foliage Litter of Rhododendron capitatum and Preparation of Aqueous Extract

The foliage litter of *R. capitatum* were collected in early September in Zhuaxixiulong Township of Tianzhu County in Gansu Province (latitude 37°11′~37°13′ N, longitude 102°23′~102°29′ E, elevation 3000~3500 m above sea level). The region belongs to the eastern edge of the Qinghai–Tibet Plateau, with alpine meadows being the dominant grassland type. *R. capitatum* is one of the main shrubs in the region, and the herbaceous plants under the shrub mainly include *Carex alatauensis*, *E. nutans*, *P. pratensis*, *Bistorta vivipara*, and *M. ruthenica*.

*R. capitatum* foliage litter was brought back to the laboratory, and 50 g of air-dried litter was placed in 500 mL of distilled water, sonicated for 30 min and then soaked for 24 h at 25 °C [32,44]. The extract was passed through 2 layers of cheesecloth to remove all solid substances and then filtered using 0.45 µm filter paper to obtain the original solution with a concentration of 100 mg·mL^−1^, which was stored at 4 °C until use. The stock solution was diluted with distilled water to obtain concentrations of 12.5, 25, 50, 75, and 100 mg·mL^−1^ before use.

### 4.2. Extraction of Compounds from Foliage Litter of Rhododendron capitatum

An amount of 25 mg of litter samples were placed in a centrifuge tube, and 500 μL of extraction solutions were added (MeOH:ACN:H_2_O = 2:2:1 (*v*/*v*); the solutions contain deuterated internal standards), and the mixed solution was vortexed for 30 s. Then, the mixed sample was homogenized (35 Hz, 4 min) and sonicated for 5 min in 4 °C water baths, and this was repeated three times. The sample was incubated for 1 h at −40 °C to precipitate proteins. Then, the samples were centrifuged at 12,000 rpm for 15 min at 4 °C. The supernatant was transferred to a fresh glass vial for analysis.

### 4.3. LC-MS/MS Analysis

LC-MS/MS (Liquid Chromatograp-Mass Spectrometry/Mass Spectrometry) analyses were performed using an UHPLC system (Vanquish, Thermo Fisher Scientific, Waltham, MA, USA) with a Phenomenex Kinetex C18 (2.1 mm × 50 mm, 2.6 μm) coupled to Orbitrap Exploris 120 mass spectrometer (Orbitrap MS, Thermo Fisher Scientific, MA, USA). The mobile phase A was 0.01% acetic acid in water; mobile phase B was IPA:ACN (1:1, *v*/*v*). The auto-sampler temperature was 4 °C, and the injection volume was 2 μL. The Orbitrap Exploris 120 mass spectrometer was used for its ability to acquire MS/MS spectra in information-dependent acquisition (IDA) mode in the control of the acquisition software (Xcalibur, Thermo Fisher Scientific, MA, USA). Annotated structures were assigned with different confidence levels according to the definition by Metabolomics Standards Initiative (MSI) [45]. Level 1: metabolites annotated using in-house metabolite standards with three orthogonal properties (i.e., MS1 + RT (retention time) + MS/MS), MS1 *m*/*z* were obtained; level 2: metabolites annotated using in-house metabolite standards with two orthogonal properties (i.e., MS1 + MS/MS) were obtained; level 3.1: known metabolites annotated with MS1, predicted RT, and surrogate MS/MS spectra (i.e., MS1 + predicted RT + surrogate MS/MS) were obtained; level 3.2: unknown structures annotated with MS1, predicted RT, and surrogate MS/MS spectra (i.e., MS1 + predicted RT + surrogate MS/MS) [46] were obtained. The raw data were converted into mzXML format using ProteoWizard software v.3.0.9134 (University of Washington, Seattle, WA, USA), and R software v.4.1.3 (XCMS package) (R Core Team, Auckland, New Zealand) was used for peak detection, extraction, alignment, and integration. Peak detection includes metabolite annotation, and the peak table was processed and generated using XCMS. The peaks of the same metabolite were aligned to the same retention time window to eliminate retention time drift between different samples through XCMS. Then, metabolites were identified by matching MS, MS/MS spectra with standard RT values in an in-house chemical standard library, and the relative content of identified metabolites was calculated by comparing the peak integration area with the in-house standard samples [47,48].

### 4.4. Determination of Allelopathic Effects of Aqueous Extracts and Compounds from Rhododendron capitatum on Elymus nutans, Poa pratensis, and Medicago ruthenica

#### 4.4.1. Allelopathy of Aqueous Extracts of *Rhododendron capitatum* Foliage Litter

Two layers of filter paper were placed in a Petri dish with a diameter of 9 cm, and 30 surface-sterilized seeds of *E. nutans*, *P. pratensis*, and *M. ruthenica* were placed in each Petri dish. The *M. ruthenica* seeds were sanded by sandpaper to break physical dormancy. The three tested seeds were provided by Gansu Agricultural University. To test the allelopathic effect, 5 mL of the foliage litter extract of *R. capitatum* or distilled water was added to Petri dish, with 3 replicates set for each treatment, for a total of 54 Petri dishes (6 concentrations (0, 12.5, 25, 50, 75, and 100 mg·mL^−1^) × 3 tested seeds × 3 replicates). Petri dishes were sealed with a sealing membrane to prevent moisture loss [44]. Next, the Petri dishes were placed in an incubator at a temperature of 20 °C for a 16/8 h (light/dark cycle), light intensity of 3500 lx, and relative humidity 60% [32]. After the third day, 2 mL of extract and distilled water were added daily to each dish. The number of germinated seeds (radius broke through the seed coat and reached 2 mm in length) was recorded every 24 h. On the tenth day of germination, the GP of seeds was measured, and 5 seedlings were randomly selected from each dish to measure SL and RL, the GI and RI was calculated. The SL and Gl of treatments without seed germination were considered as zero.

#### 4.4.2. Allelopathy of Compounds from *Rhododendron capitatum* Foliage Litter

Organic acids, fatty acids and derivatives, terpenes, phenols, and phenolic acid compounds were detected from *R. capitatum* foliage litter. The zanamivir, alpha linolenic acid, Kaurenoic acid, 4-hydroxyphenylglycolic acid, and Quinic acid, which had the highest relative content among the five types of compounds, were selected as allelopathic substances to test allelopathy. Standard samples of 4-hydroxyphenylglycolic acid were purchased from Shanghai Macklin Biochemical Technology Co., Ltd. (Shanghai, China. https://www.macklin.cn), and the standard samples of zanamivir, alpha linolic acid, Kaurenoic acid, and Quinic acid were provided by Shanghai Yuanye Bio Technology Co., Ltd. (Shanghai, China. https://www.shyuanye.com); the purity of the five samples was 98%.

The five standard samples of allelopathic substances were configured into solutions with concentrations of 0.25, 0.5, 0.75, and 1 mg·mL^−1^, respectively. Then, 30 surface-sterilized seeds of *E. nutans*, *P. pratensis*, and *M. ruthenica* were placed in Petri dishes with a diameter of 9 cm and two layers of filter paper, respectively. Five milliliters of each compound and concentration of allelopathic solution or distilled water were added to dishes, and each treatment was replicated 3 times for a total of 189 dishes (5 compounds × 4 concentrations × 3 tested seeds × 3 replicates + 3 distilled water replicates × 3 tested seeds). Then, the Petri dishes were placed in an incubator with pre-set parameters; seedling and root lengths were determined on the tenth day of germination, and GP, GI, and RI were calculated according to the following Equations (1)–(3) [37,49,50]:

GP = N_10_/N_total_ × 100% (1)

(2) $$\mathrm{GI}=(N_{1})\times 1+(N_{2}-N_{1})\times \frac{1}{2}+(N_{3}-N_{2})\times \frac{1}{3}+\ldots +(N_{n}-N_{n-1})\times \frac{1}{n}$$

RI = 1 − C/T (T ≥ C) or T/C − 1 (T < C)(3)

where N_10_ is the number of germinated seeds on day 10; N_total_ is the total number of seeds for the test; N_1_, N_2_, N_3_, …, N_n_ is the proportion of germinated seeds observed after 1, 2, 3, …, n days; C is the corresponding value for the Control; T is the treatment value. Positive values of RI show a stimulatory effect, and negative ones indicate inhibitory activity of the aqueous extract.

### 4.5. Statistical Analysis

All data were analyzed using SPSS 24.0 (SPSS Inc., Chicago, IL, USA) and presented as mean ± standard error. One-way analysis of variance (ANOVA) and Tukey’s multiple comparisons were used to test the differences between treatments. Figures were created with OriginPro 2022 (Origin Lab Corporation, Northampton, MA, USA).

## 5. Conclusions

The aqueous extract of the foliage litter of *R. capitatum* had an allelopathy of “low concentration promotion and high concentration inhibition” on the germination of *E. nutans*, *P. pratensis*, and *M. ruthenica* seed, and the threshold concentration for inhibiting the germination of *E. nutans* and *P. pratensis* was 25 mg·mL^−1^. Organic acids, fatty acids, terpenes, phenols, and phenolic acid compounds were detected in the foliage litter of *R. capitatum*, and Zanamivir, alpha-Lipogenic acid, Kaurenoic acid, 4-hydroxyphenylglycolic acid, and Quinic acid were the five compounds with the highest relative content. The standard addition test showed that the five compounds had inhibitory allelopathy on the seed germination and seedling growth of *E. nutans*, *P. pratensis*, and *M. ruthenica*. Higher concentrations of alpha-Linolic acid, Kaurenoic acid, and 4-hydroxyphenylglycolic acid completely inhibited the germination of *P. pratensis*. This study further elucidated the negative allelopathy and allelochemicals of *R. capitatum* on perennial herbaceous plants.

## Figures and Tables

**Table 1:** Relative content of organic acid compounds extracted from foliage litter of *Rhododendron capitatum*.

Number	Retention Time (s)	Name	Relative Content (%)	Molecular Formula
1	201.6	Zanamivir	77.81	C_12_H_20_N_4_O_7_
2	48.3	Citric acid	9.57	C_6_H_8_O_7_
3	95.3	Allicin	3.39	C_6_H_10_OS_2_
4	325.3	Fumoni + sin A1	3.04	C_36_H_61_NO_16_
5	163	Dopamine 3-O-sulfate	2.03	C_8_H_11_NO_5_S
6	138	Chicoric acid	1.29	C_22_H_18_O_12_
7	192.2	Auranofin	1.19	C_20_H_34_AuO_9_PS
8	63.6	Carboplatin	1.01	C_6_H_12_N_2_O_4_Pt

This table only lists compounds with a relative content > 1%. The same applies to Table 2, Table 3, Table 4 and Table 5.

**Table 2:** Relative content of fatty acids and derivatives compounds extracted from foliage litter of *Rhododendron capitatum*.

Number	Retention Time (s)	Name	Relative Content (%)	Molecular Formula
1	301.5	alpha-linolenic acid	18.00	C_18_H_30_O_2_
2	161.7	2-Hydroxyhexanoic acid	9.05	C_6_H_12_O_3_
3	24.8	cis-Aconitic acid	6.61	C_6_H_6_O_6_
4	261.1	Pelargonic acid	5.31	C_9_H_18_O_2_
5	214.5	2-Hexyl-5-[2-(4-hydroxy-3-methoxyphenyl)ethyl]furan	4.36	C_19_H_26_O_3_
6	320.4	Palmitic acid	3.79	C_16_H_32_O_2_
7	311	Linoleic acid	3.79	C_18_H_32_O_2_
8	24.9	2-methylcitrate	3.00	C_7_H_10_O_7_
9	335.7	Erucamide	2.94	C_22_H_43_NO
10	25.2	2-Hydroxyglutaric acid	2.85	C_5_H_8_O_5_
11	216.7	Caproic acid	2.17	C_6_H_12_O_2_
12	223.4	Oxalic acid	1.87	C_2_H_2_O_4_
13	197.3	Azelaic acid	1.80	C_9_H_16_O_4_
14	47.1	2-Hydroxyhexanedioic acid	1.74	C_6_H_10_O_5_
15	145.8	Hydroxyisocaproic acid	1.49	C_6_H_12_O_3_
16	321.9	Oleic acid	1.30	C_18_H_34_O_2_
17	53.2	Glutaric acid	1.23	C_5_H_8_O_4_
18	101.6	Ketoleucine	1.18	C_6_H_10_O_3_
19	306.1	Myristic acid	1.12	C_14_H_28_O_2_
20	228.8	Undecanedioic acid	1.08	C_11_H_20_O_4_

**Table 3:** Relative content of terpenoids compounds extracted from foliage litter of *Rhododendron capitatum*.

Number	Retention Time (s)	Name	Relative Content (%)	Molecular Formula
1	237.4	Kaurenoic acid	23.50	C_20_H_30_O_2_
2	282.9	2-Angeloyl-9-(3-methyl-2E-pentenoyl)-2b,9a-dihydroxy-4Z,10(14)-oplopadien-3-one	10.98	C_26_H_36_O_5_
3	258.7	Arjunolic acid	8.44	C_30_H_48_O_5_
4	286.7	Ceanothic acid	5.74	C_30_H_46_O_5_
5	305	beta-Boswellic acid	5.23	C_30_H_48_O_3_
6	321.6	3-cis-Hydroxy-b,e-Caroten-3′-one	4.69	C_40_H_54_O
7	200.4	Xanthorrhizol	4.47	C_15_H_22_O
8	232.1	(R)-ar-Turmerone	3.35	C_15_H_20_O
9	316.8	3-Hydroxy-b,e-caroten-3′-one	2.61	C_40_H_54_O_2_
10	226.7	Gibberellin A79	2.60	C_19_H_24_O_7_
11	263.8	Steviol	2.10	C_20_H_30_O_3_
12	292.8	Auroxanthin	1.83	C_40_H_56_O_4_
13	155.9	Gossypol	1.61	C_30_H_30_O_8_
14	269.2	2-(2-Methylbutanoyl)-9-(3-methyl-2E-pentenoyl)-2b,9a-dihydroxy-4Z,10(14)-oplopadien-3-one	1.24	C_26_H_38_O_5_
15	291.2	Masticadienonic acid	1.23	C_30_H_46_O_3_
16	280.6	18-alpha-Glycyrrhetinic acid	1.11	C_30_H_46_O_4_
17	305.3	Betulinic acid	1.11	C_30_H_48_O_3_
18	307.2	Mutatoxanthin	1.05	C_40_H_56_O_3_
19	295.6	Idoxanthin	1.01	C_40_H_54_O_4_

**Table 4:** Relative content of phenols compounds extracted from foliage litter of *Rhododendron capitatum*.

Number	Retention Time (s)	Name	Relative Content (%)	Molecular Formula
1	37.1	4-Hydroxyphenylglycolic acid	21.54	C_8_H_8_O_4_
2	51.7	Epinephrine	17.67	C_9_H_13_NO_3_
3	224.4	6-Hydroxyshogaol	15.49	C_17_H_24_O_4_
4	141.8	4-Methylcatechol	13.36	C_7_H_8_O_2_
5	204.3	2-Methoxy-4-propylphenol	10.30	C_10_H_14_O_2_
6	238.2	[10]-Gingerdione	9.74	C_21_H_32_O_4_
7	215.6	Capsiate	8.58	C_18_H_26_O_4_
8	145.8	3,4-Dimethylphenol	2.77	C_8_H_10_O

**Table 5:** Relative content of phenolic acid compounds extracted from foliage litter of *Rhododendron capitatum*.

Number	Retention Time (s)	Name	Relative Content (%)	Molecular Formula
1	22.3	Quinic acid	28.24	C_7_H_12_O_6_
2	208.4	Methyl vanillate	18.13	C_9_H_10_O_4_
3	232.6	Propyl paraben	12.20	C_10_H_12_O_3_
4	322.3	DEHP	9.59	C_24_H_38_O_4_
5	214.8	2,6-Dimethoxybenzoic acid	4.24	C_9_H_10_O_4_
6	157.8	Methylgallate	3.82	C_8_H_8_O_5_
7	187.6	Propylgallate	2.29	C_10_H_12_O_5_
8	70.4	3,5-Dihydroxybenzoic acid	2.04	C_7_H_6_O_4_
9	143.2	Salicin	1.84	C_13_H_18_O_7_
10	148.8	Salicylic acid	1.83	C_7_H_6_O_3_
11	27.5	Arbutin	1.83	C_12_H_16_O_7_
12	146.3	Acetovanillone	1.75	C_9_H_10_O_3_
13	173.2	Hydroferulic acid	1.72	C_10_H_12_O_4_
14	215.4	Ethyl 4-hydroxybenzoate	1.67	C_9_H_10_O_3_
15	84.9	Gentisic acid	1.57	C_7_H_6_O_4_
16	110.7	3-methylolphenol	1.17	C_7_H_8_O_2_
17	168.5	Vanillin	1.10	C_8_H_8_O_3_
18	166.5	2,6-Dihydroxybenzoic acid	1.06	C_7_H_6_O_4_
19	156.1	Homoveratric acid	1.00	C_10_H_12_O_4_

## Data Availability

The data presented in this study are available on request from the corresponding author. The data are not publicly available due to privacy.

## Supplementary Materials

The following supporting information can be downloaded at: https://www.mdpi.com/article/10.3390/plants13182585/s1, Table S1: Allelopathic response index of water extracts from *Rhododendron capitatum* foliage litter on the germination and growth of *Elymus nutans*, *Poa pratensis* and *Medicago ruthenica*; Table S2: Allelopathic response index of five components from *Rhododendron capitatum* on the germination and growth of *Elymus nutans*, *Poa pratensis* and *Medicago ruthenica*.
