# Supplementary material for: Isolation of Allelochemicals from Rhododendron capitatum and Their Allelopathy on Three Perennial Herbaceous Plants
Source: Plants (Basel). 2024 Sep 15;13(18):2585. doi: 10.3390/plants13182585 (PMC11434890; doi:10.3390/plants13182585)
Supplement: Supplementary file 1 [file plants-13-02585-s001.zip › plants-3198967-supplementary.pdf]

Table S1. Allelopathic response index of water extracts from *Rhododendron capitatum* foliage litter on the germination and growth of *Elymus nutans*, *Poa pratensis* and *Medicago ruthenica*.

Concentration (mg·mL⁻¹)	*E. nutans*				*P. pratensis*				*M. ruthenica*			
	GP	GI	SL	RL	GP	GI	SL	RL	GP	GI	SL	RL
12.5	0.01± 0.03b	0.02± 0.02ab	0.17± 0.03a	0.02± 0.05a	0.27± 0.03a	0.25± 0.03a	0.05± 0.02ab	0.06± 0.06a	0.09± 0.04ab	0.06± 0.03b	0.02± 0.02a	-0.01± 0.02a
25	0.08± 0.02a	0.06± 0.02a	0.10± 0.02a	0.04± 0.02a	0.35± 0.03a	0.33± 0.01a	0.10± 0.05a	0.00± 0.02ab	0.11± 0.02a	0.10± 0.03ab	0.01± 0.03a	-0.02± 0.05ab
50	0.03± 0.04ab	0.03± 0.02a	0.08± 0.03ab	0.04± 0.05a	-0.06± 0.04bc	-0.04± 0.01b	-0.03± 0.02b	-0.05± 0.02b	0.12± 0.02a	0.21± 0.04a	0.06± 0.04ab	-0.03± 0.03ab
75	-0.10± 0.05bc	-0.07± 0.04ab	-0.01± 0.05b	-0.02± 0.05a	-1.00± 0.00c	-1.00± 0.00c	-1.00± 0.00c	-1.00± 0.00c	-0.07± 0.05b	0.02± 0.03bc	-0.03± 0.02a	-0.10± 0.04ab
100	-0.28± 0.03c	-0.19± 0.02b	-0.03± 0.05ab	-0.19± 0.08b-	-1.00± 0.00c	-1.00± 0.00c	-1.00± 0.00c	-1.00± 0.00c	-0.12± 0.05b	-0.11± 0.07c	-0.08± 0.03a	-0.19± 0.01b

Experimental data are expressed as mean ± standard error (n = 3). Different lowercase letters indicate a significant difference at $P < 0.05$. GP represents germination percentage; GI represents germination index; SL represents shoot length; RL represents root length.

Table S2. Allelopathic response index of five components from *Rhododendron capitatum* on the germination and growth of *Elymus nutans*, *Poa pratensis* and *Medicago ruthenica*.

**Compound**	**Concentration ($mg\cdot mL^{-1}$)**	***E. nutans***				***P. pratensis***				***M. ruthenica***			
		**GP**	**GI**	**SL**	**RL**	**GP**	**GI**	**SL**	**RL**	**GP**	**GI**	**SL**	**RL**
Z	0.25	-0.03±0.03a	-0.08±0.02a	-0.07±0.06a	-0.20±0.06a	-0.31±0.06a	-0.4±0.06a	-0.01±0.05a	-0.06±0.06a	-0.42±0.12a	-0.57±0.09a	0.1±0.04a	-0.46±0.06a
	0.50	-0.11±0.06a	-0.28±0.04b	-0.32±0.03b	-0.36±0.03ab	-0.47±0.03a	-0.56±0.02ab	-0.26±0.05b	-0.17±0.07ab	-0.66±0.02ab	-0.69±0.01ab	0.03±0.08a	-0.47±0.07a
	0.75	-0.02±0.02a	-0.29±0.02b	-0.48±0.03c	-0.50±0.04b	-0.59±0.08a	-0.69±0.06b	-0.52±0.05c	-0.56±0.03c	-0.81±0.03b	-0.82±0.03b	-0.21±0.09a	-0.70±0.04b
	0.10	-0.05±0.01a	-0.32±0.04b	-0.37±0.03bc	-0.43±0.03b	-0.55±0.07a	-0.64±0.07ab	-0.34±0.06bc	-0.35±0.05bc	-0.75±0.05b	-0.79±0.03ab	-0.15±0.10a	-0.61±0.03ab
aLa	0.25	-0.06±0.01a	-0.27±0.02a	-0.38±0.03a	-0.30±0.05a	-0.48±0.03a	-0.58±0.03a	-0.22±0.07a	-0.22±0.06a	-0.46±0.04a	-0.60±0.04a	-0.13±0.05a	-0.42±0.03a
	0.50	-0.06±0.03a	-0.35±0.05ab	-0.40±0.05a	-0.38±0.06a	-1.00±0.00b	-1.00±0.00b	-1.00±0.00b	-1.00±0.00b	-0.80±0.05b	-0.81±0.06a	-0.37±0.06b	-0.52±0.04ab
	0.75	-0.15±0.04a	-0.45±0.04b	-0.44±0.03a	-0.46±0.03a	-1.00±0.00b	-1.00±0.00b	-1.00±0.00b	-1.00±0.00b	-0.73±0.06b	-0.76±0.07a	-0.16±0.08ab	-0.42±0.03a
	0.10	-0.07±0.02a	-0.37±0.01ab	-0.47±0.03a	-0.30±0.03a	-1.00±0.00b	-1.00±0.00b	-1.00±0.00b	-1.00±0.00b	-0.76±0.02b	-0.80±0.00a	-0.20±0.04ab	-0.62±0.03b
Ka	0.25	-0.06±0.03a	-0.31±0.06a	-0.34±0.06a	-0.28±0.06a	-0.66±0.09a	-0.71±0.07a	-0.15±0.06a	-0.21±0.05a	-0.47±0.10a	-0.58±0.07a	0.02±0.03a	-0.31±0.08a
	0.50	-0.04±0.02a	-0.30±0.01a	-0.45±0.02ab	-0.36±0.04a	-0.83±0.02ab	-0.88±0.01b	-0.67±0.03b	-0.62±0.03b	-0.59±0.05a	-0.63±0.02a	0.06±0.05a	-0.69±0.02b
	0.75	-0.53±0.05b	-0.69±0.04b	-0.59±0.04b	-0.66±0.07b	-1.00±0.00b	-1.00±0.00b	-1.00±0.00b	-1.00±0.00b	-1.00±0.00b	-1.00±0.00b	-1.00±0.00b	-1.00±0.00b
	0.10	-0.63±0.04b	-0.78±0.02b	-0.77±0.03c	-0.79±0.04b	-1.00±0.00b	-1.00±0.00b	-1.00±0.00b	-1.00±0.00b	-1.00±0.00b	-1.00±0.00b	-1.00±0.00b	-1.00±0.00b

Table S2. *Cont.*

Compound	Concentration (mg·mL^{-1})	*E. nutans*				*P. pratensis*				*M. ruthenica*			
		GP	GI	SL	RL	GP	GI	SL	RL	GP	GI	SL	RL
4Ha	0.25	-0.04± 0.02a	-0.26± 0.02a	-0.27± 0.03a	-0.31± 0.06a	-0.48± 0.05a	-0.56± 0.03a	-0.5± 0.08a	-0.5± 0.07a	-0.42± 0.03a	-0.47± 0.03a	-0.02± 0.05a	-0.42± 0.04a
	0.50	-0.14± 0.03ab	-0.42± 0.03b	-0.52± 0.03b	-0.56± 0.02b	-0.59± 0.06a	-0.64± 0.05a	-0.39± 0.11a	-0.41± 0.08a	-0.49± 0.08a	-0.52± 0.06ab	-0.16± 0.05ab	-0.57± 0.02b
	0.75	-0.16± 0.01b	-0.53± 0.01c	-0.64± 0.03b	-0.67± 0.02b	-1.00± 0.00b	-1.00± 0.00b	-1.00± 0.00b	-1.00± 0.00b	-0.71± 0.03b	-0.67± 0.02b	-0.18± 0.03b	-0.62± 0.03b
	0.10	-0.38± 0.03c	-0.58± 0.03c	-0.64± 0.05b	-0.64± 0.05b	-1.00± 0.00b	-1.00± 0.00b	-1.00± 0.00b	-1.00± 0.00b	-1.00± 0.00b	-1.00± 0.00b	-1.00± 0.00b	-1.00± 0.00b
Qa	0.25	-0.01± 0.03a	-0.15± 0.04a	-0.24± 0.03a	-0.2± 0.04b	-0.38± 0.06a	-0.41± 0.06a	-0.04± 0.05b	-0.11± 0.05bc	-0.37± 0.02a	-0.39± 0.04a	0.24± 0.02a	-0.14± 0.09a
	0.50	-0.04± 0.00a	-0.03± 0.02a	-0.16± 0.03a	-0.11± 0.06ab	-0.28± 0.05a	-0.26± 0.04a	0.15± 0.04a	0.15± 0.03a	-0.34± 0.08a	-0.36± 0.06a	0.22± 0.04ab	-0.17± 0.08a
	0.75	-0.02± 0.01a	-0.09± 0.03a	-0.24± 0.01a	0.01± 0.04a	-0.28± 0.1a	-0.29± 0.09a	-0.04± 0.04b	0.02± 0.06ab	-0.34± 0.03a	-0.33± 0.02a	0.09±0.05b	-0.16± 0.1a
	0.10	0.00± 0.02a	-0.1± 0.04a	-0.2± 0.03a	-0.09± 0.04ab	-0.4± 0.05a	-0.48± 0.05a	-0.11± 0.04b	-0.23± 0.07c	-0.37± 0.08a	-0.44± 0.08a	0.18± 0.03ab	-0.23± 0.08a

Experimental data are expressed as mean ± standard error (n = 3). Different lowercase letters indicate a significant difference at $P < 0.05$. GP represents germination percentage; GI represents germination index; SL represents shoot length; RL represents root length.
